# First person – Craig Keenan

**DOI:** 10.1242/dmm.046193

**Published:** 2020-07-14

## Abstract

First Person is a series of interviews with the first authors of a selection of papers published in Disease Models & Mechanisms, helping early-career researchers promote themselves alongside their papers. Craig Keenan is first author on ‘Post-traumatic osteoarthritis development is not modified by postnatal chondrocyte deletion of *Ccn2*’, published in DMM. Craig conducted the research described in this article while a postdoctoral research associate in Dr Blandine Poulet's lab at the University of Liverpool, Liverpool, UK. He is now a lecturer in vertebrate physiology in the lab of Dr Jason Kirby at Liverpool John Moores University, Liverpool, UK, investigating the roles of cartilage and bone in the pathogenesis of degenerative joint disease.


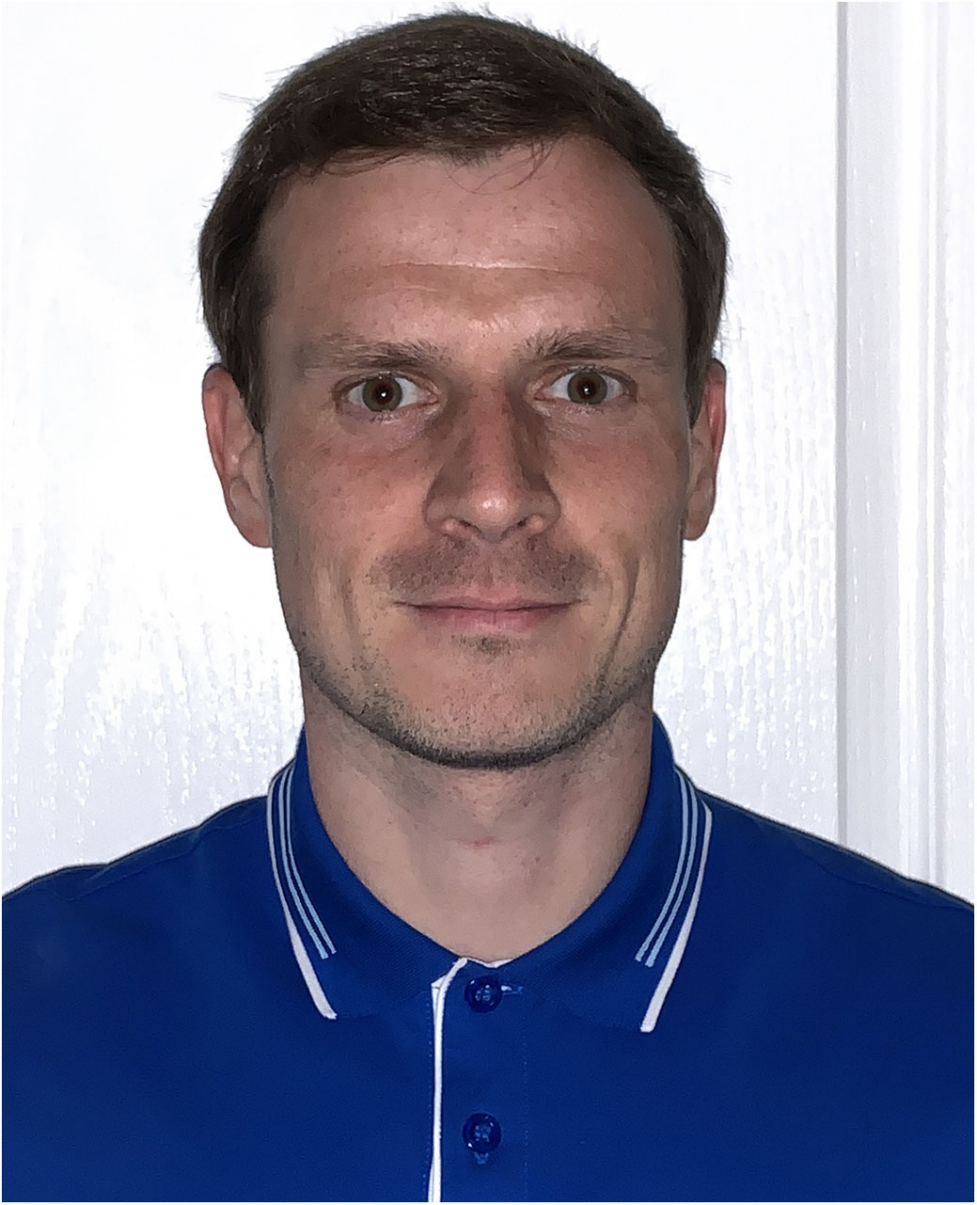


**Craig Keenan**

**How would you explain the main findings of your paper to non-scientific family and friends?**

Our research focused on a protein known as CCN2. CCN2 is critical in the development of cartilage, a tissue that covers and protects the ends of long bones. In ageing and/or following injuries, cartilage can become damaged and lead to the development of a disease known as osteoarthritis (OA). The role of CCN2 in adult cartilage and OA is currently unclear, with both positive and negative results being reported in the literature. Therefore, we wanted to determine the role of CCN2 explicitly in adult cartilage, by removing CCN2 from all cartilage cells (chondrocytes) and observing how this affected the development of OA, in two different mouse models of injury-induced OA. We found no effects of removing CCN2 in cartilage, suggesting that the presence of CCN2 in adult chondrocytes is not critical in protecting cartilage from OA-related degeneration.

**What are the potential implications of these results for your field of research?**

Although we know from other studies that CCN2 is important in OA, we now recognize that this is not linked to the production of this protein from cartilage cells. The roles of other cells present in the knee joint appear to be more important than first thought and need more investigation.

**What are the main advantages and drawbacks of the model system you have used as it relates to the disease you are investigating?**

Transgenic mice, in which the genome has been manipulated, have become an almost indispensable tool for studying the development of diseases. The main advantage of using them in our study was that we were able to generate conditional *Ccn2* knockout mice in which *Ccn2* was specifically deleted from chondrocytes. In addition, we used two distinct models of OA induced by either surgical injury or repetitive mechanical loading. These have previously been used to mimic some aspects of human OA. The main drawbacks come from the fact that mice show important differences to humans and studies using them need to be interpreted carefully.

**What has surprised you the most while conducting your research?**

The sheer number of samples and histology sections that require analysis when conducting an *in vivo* study in joints!

“[…] increasing the number of pilot/seed grants offered by funders would provide an ideal opportunity for early-career scientists to get their foot on the ladder and establish themselves as an independent academic.”

**Describe what you think is the most significant challenge impacting your research at this time and how will this be addressed over the next 10 years?**

Funding. It is becoming increasingly difficult to secure funding and, as an early-career scientist trying to establish my own independent research profile, this is very concerning. I believe funders should do more to make sure funding is distributed across a wide spectrum and not only to established researchers. I think increasing the number of pilot/seed grants offered by funders would provide an ideal opportunity for early-career scientists to get their foot on the ladder and establish themselves as an independent academic.

**Figure:**
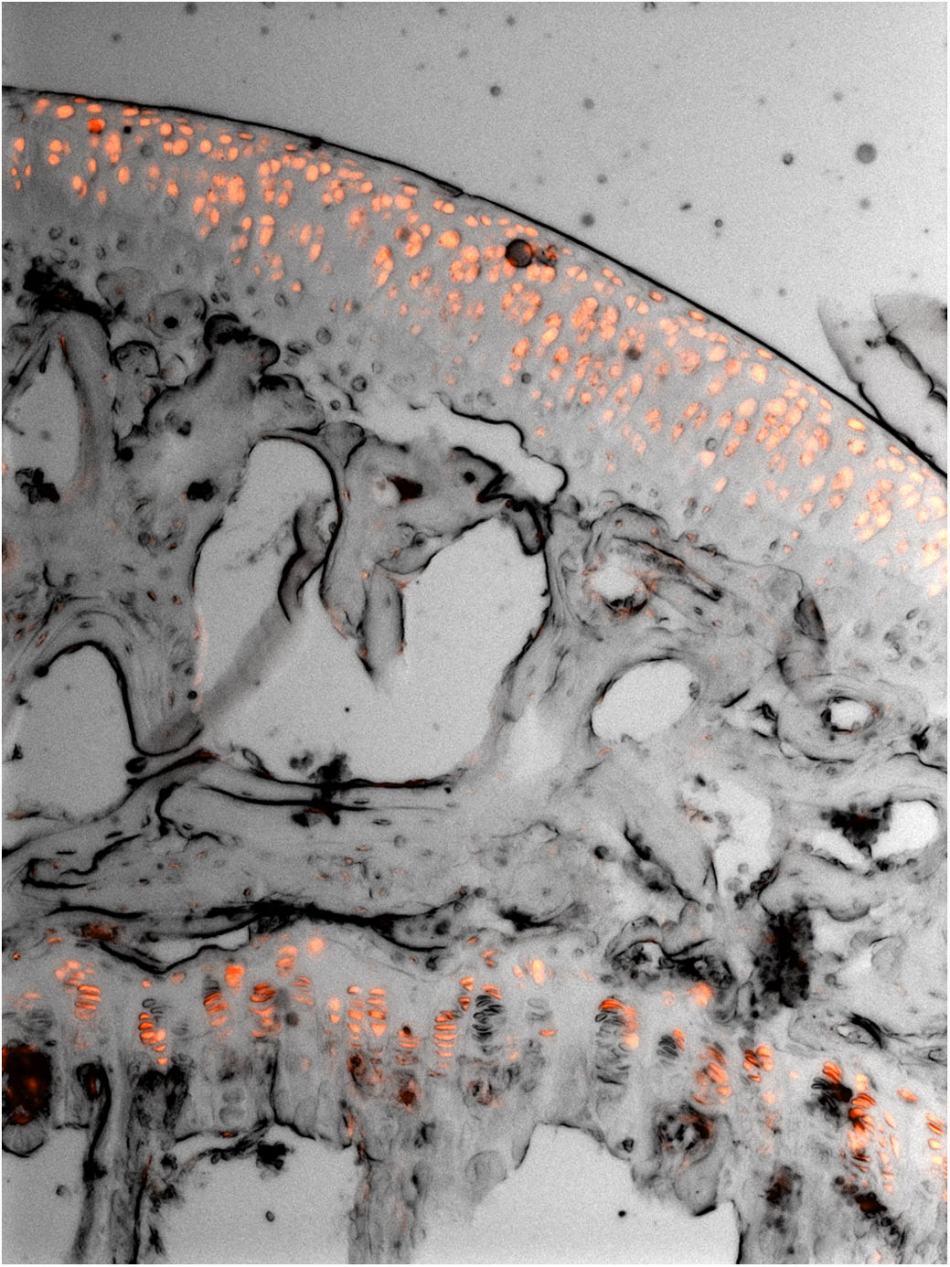
**Expression of tdTomato fluorescent protein in chondrocytes of mouse articular (top) and growth plate (bottom) cartilage – the protein is present in all cells from which the transgene is deleted.**

**What changes do you think could improve the professional lives of early-career scientists?**

The single, most important aspect for any early-career scientist hoping to establish themselves is to be given the opportunity to progress from postdoctoral to faculty level. Unfortunately, the lack of available positions is a problem that has long affected academia. Postdoc positions are great as they help increase your research experience/output, but they are only ever temporary and very rarely lead to the offer of a permanent position. This uncertainty can be highly stressful and can ultimately lead to highly talented scientists questioning whether academia is the right fit for them. I believe it is vitally important that postdocs are provided with all the necessary tools required to transition to the next stage of their career and that ample job opportunities are provided to make this a reality.

**What's next for you?**

I was recently (February 2020) appointed to the position of lecturer in vertebrate physiology at Liverpool John Moores University. This marks the start of my (hopefully successful) transition from postdoc researcher to independent academic and is something I have worked towards for a long time.
